# Benefits of aflibercept treatment for age-related macular degeneration patients with good best-corrected visual acuity at baseline

**DOI:** 10.1038/s41598-017-18255-4

**Published:** 2018-01-08

**Authors:** Sakiko Minami, Norihiro Nagai, Misa Suzuki, Toshihide Kurihara, Hideki Sonobe, Mamoru Kamoshita, Atsuro Uchida, Hajime Shinoda, Hitoshi Takagi, Shozo Sonoda, Taiji Sakamoto, Kazuo Tsubota, Yoko Ozawa

**Affiliations:** 1 Department of Ophthalmology, Keio University School of Medicine, Tokyo, Japan; 2 Department of Ophthalmology, St. Marianna University School of Medicine, Kanagawa, Japan; 3 Inagi Municipal Hospital, Tokyo, Japan; 4 Department of Ophthalmology, Kagoshima University Graduate School of Medical and Dental Sciences, Kagoshima, Japan; 5 Laboratory of Retinal Cell Biology, Keio University School of Medicine, Tokyo, Japan

## Abstract

Currently, age-related macular degeneration (AMD) is treated while patients exhibit good best-corrected visual acuity (BCVA). However, previous clinical trials only include patients with poor BCVA. We prospectively analyzed the benefits of intravitreal aflibercept (IVA) treatment for AMD patients exhibiting good BCVA at baseline. Twenty-nine treatment-naive AMD patients (29 eyes) with BCVA better than 0.6 (74 letters in ETDRS chart) were treated with IVA once a month for 3 months and every 2 months thereafter with no additional treatments. Improvement in mean BCVA, measured using the conventional Landolt C chart, contrast VA chart, and functional VA (FVA) system, and reductions in mean central retinal thickness (CRT), central choroidal thickness, macular volume (MV), and choroidal area on optical coherence tomography images were observed at 6 and 12 months. Improvements in contrast VA and FVA scores, in contrast to conventional BCVA, correlated with MV reduction; no VA scores correlated with a reduced CRT. The MV correlated with choroidal area after IVA. No severe adverse events occurred. IVA improved visual function, retinal condition, and quality of life evaluated by Visual Function Questionnaire, and was beneficial in these patients. The contrast VA and FVA scores and MVs, which detect subtle changes, helped demonstrate the benefits.

## Introduction

Age-related macular degeneration (AMD) is one of the leading causes for blindness worldwide, although several anti-vascular endothelial growth factor (anti-VEGF) therapies are widely available. Blindness can be partly attributed to irreversible retinal damage after AMD onset. Therefore, patients with AMD should be treated early, before a deterioration in their best-corrected visual acuity (BCVA). However, previous prospective clinical studies^1–4^ included patients with a score of 73 to 25 letters on the Early Treatment Diabetic Retinopathy Study (ETDRS) visual acuity (VA) chart or its Snellen equivalent, 20/40 to 20/320. The CATT study^5^ was designed for patients with a Snellen BCVA ranging from 20/25 to 20/320 and included better BCVA than the other trials^1–4^. However, few prospective clinical studies have been designed only for patients with a good BCVA at baseline who may exhibit a ceiling effect^6,7^ and may not show an improvement after treatment. Knowledge of the therapeutic prognosis influences treatment planning and the informed consent procedure.

In addition to the Landolt C chart, which is used in daily clinical practice in Japan, contrast VA^8–10^ and functional VA (FVA)^9–11^ measurements are used to detect subtle changes in BCVA. Contrast VA is measured using the Landolt C chart showing gray letters against a white background, and low-contrast acuity measurements capture vision loss that is not observed with high-contrast measurements^8^. The FVA system is used to record BCVA in patients shown the Landolt C chart successively every 2 seconds for 1 minute with dynamic changes of the optotype size^11^. It was first used for detecting subtle BCVA changes in patients with dry eyes^12,13^, and its indications have now expanded to macular diseases such as epiretinal membrane^9^ and AMD^10^. In patients with AMD^10^, the FVA score worsened for cases that hardly showed any abnormalities in measurements obtained using the conventional Landolt C chart.

The central retinal thickness (CRT) measured in cross-sectional optical coherence tomography (OCT) images obtained through the fovea is often used to evaluate AMD^14–16^. In addition, the macular volume (MV) reflects morphological changes in the macular area, which is a more spatial region than the fovea^15,16^. A previous study showed that MV significantly improved in AMD patients without a detectable improvement in CRT^17^.

Subjective visual changes in daily life are assessed using the National Eye Institute 25-item Visual Function Questionnaire (NEI-VFQ-25), which was originally developed for analyzing health-related quality of life (QOL) and captures the influence of vision on multiple dimensions such as emotional well-being and social functioning^18^. On the basis of the English version, similar questionnaires have been developed and optimized in the Chinese^19^, Arabic^20^, and Japanese languages^21^.

In order to increase understanding of the prognosis of AMD treated before a substantial deterioration in BCVA, we prospectively analyzed the treatment effects of intravitreal aflibercept (IVA) injection in treatment-naïve patients with AMD exhibiting a good baseline BCVA (better than 0.6). Evaluations were focused on visual function, OCT findings, and VA-associated QOL.

## Results

A total of 29 eyes of 29 patients with a BCVA better than 0.6 when measured using the conventional Landolt C chart (<0.22 logMAR; better than 74 letters on the ETDRS chart) who were diagnosed with exudative AMD received IVA treatment as the first and the only treatment for AMD, according to the protocol (Table 1). The IVA injections were administered once a month for 3 months and every 2 months thereafter (2Q8 regimen). The mean age at baseline was 74.0 ± 8.76 years (range, 51 to 86 years), and 17 (59%) patients were male (Table 2). Eleven (38%) eyes exhibited typical AMD, 17 (59%) exhibited polypoidal choroidal vasculopathy (PCV), and one (3%) exhibited retinal angiomatous proliferation (RAP). From the total, 26 (90%) patients exhibited unilateral AMD. All but one eye (3%) with pseudophakia had slight to mild cataract. BCVA in the contralateral eye was better than 0.8 in 26 (90%) patients. Fourteen (48%) patients had hypertension and five (17%) had diabetes at baseline. None of the eyes were diagnosed with diabetic retinopathy.

**Table 1:** Study protocol.

								Primary Endpoint						Secondary Endpoint
Visit after initial IVA		1	2	3	4	5	6	7	8	9	10	11	12	13
Month(s) after initial IVA	Baseline	0	1	2	3	4	5	6	7	8	9	10	11	12
Preceding injection to the follow-up		○	○	○		○		○		○		○		
BCVA	○	○	○	○	○	○	○	○	○	○	○	○	○	○
OCT	○	○	○	○	○	○	○	○	○	○	○	○	○	○
FA/ICGA	○				○			○						○
Contrast VA/FVA	○				○			○						○
VFQ-25	○							○						○

IVA, intravitreal aflibercept; BCVA, best-corrected visual acuity; OCT, optical coherence tomography; FA, fluorescein angiography; ICGA, indocyanine green angiography; Contrast VA, contrast visual acuity; FVA, functional visual acuity; VFQ-25, 25-Item Visual Function Questionnaire.

**Table 2:** Baseline characteristics.

Age, years (mean ± SD)	51–86 (74.0 ± 8.76)
Male, eyes (%)	17 (59)
BCVA (mean ± SD)	0.07 ± 0.09
AMD subtypes, eyes (%)	
typical AMD	11 (38)
PCV	17 (59)
RAP	1 (3)
unilateral AMD, eyes (%)	26 (90)
Pseudophakia, eyes (%)	1 (3)
Hypertension, patients (%)	14 (48)
Diabetes, patients (%)	5 (17)

Data are shown in mean ± standard deviation. BCVA, best-corrected visual acuity; AMD, age-related macular degeneration; PCV, polypoidal choroidal vasculopathy; RAP, retinal angiomatous proliferation.

**Figure 1:**
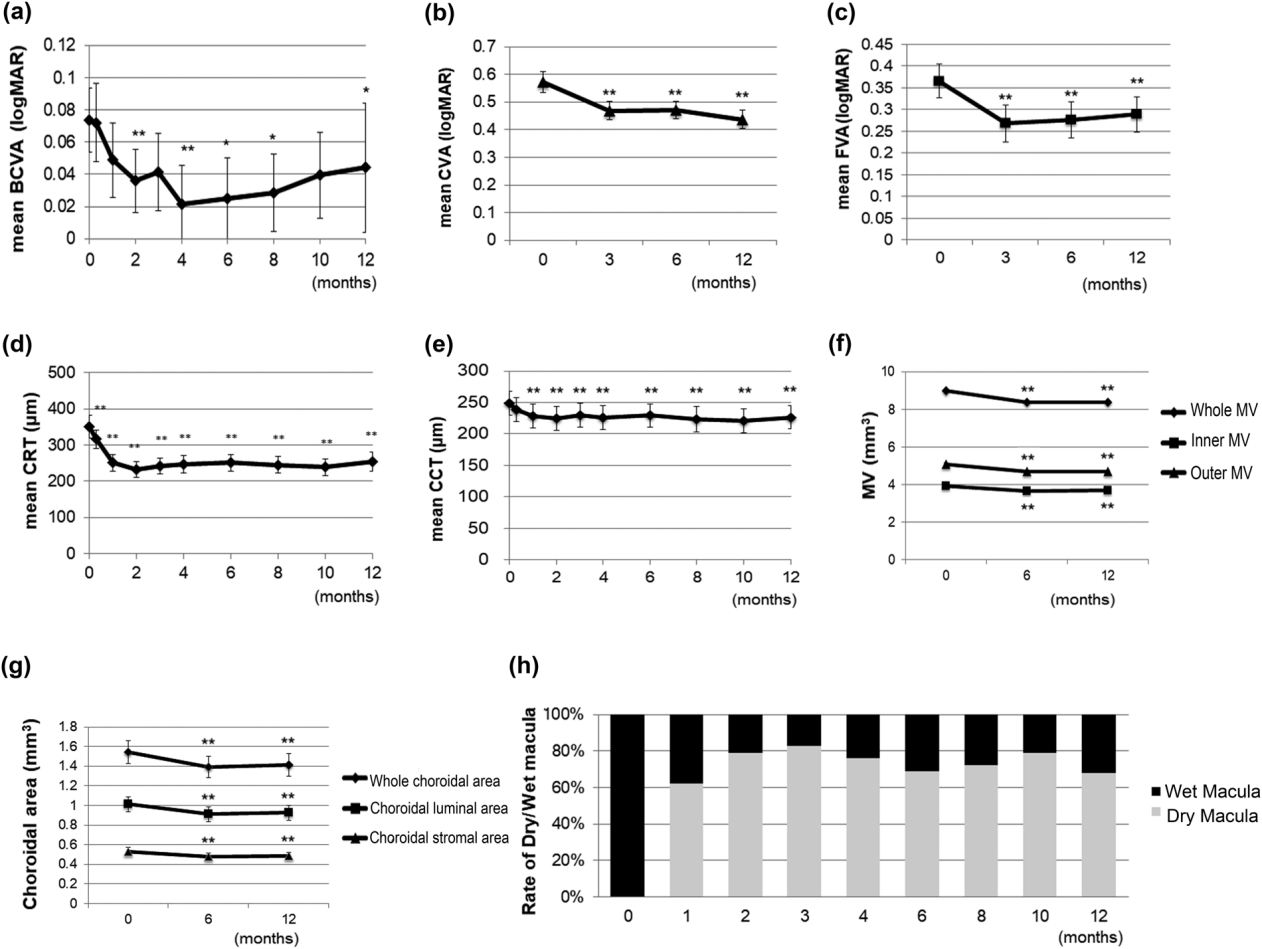
Clinical outcomes after initial intravitreal aflibercept (IVA) injections for patients with age-related macular degeneration exhibiting a good BCVA at baseline. (**a**–**g**) The mean logMAR BCVA (**a**), CVA (**b**), FVA (**c**), CRT (**d**), CCT (**e**), MV (**f**), and choroidal area (**g**) values at each time point after initial IVA treatment are plotted. (**h**) The proportion of eyes with a dry macula without exudative changes over the retinal pigment epithelium is 80%, 66% and 71% at 2, 6 and 12 months after initial IVA injections, respectively. The dry macula is shown in gray. BCVA, best-corrected visual acuity; CVA, contrast VA; FVA, functional visual acuity; CRT, central retinal thickness; CCT, central choroidal thickness; MV, macular volume. Data are shown as mean ± standard deviation. *P < 0.05, **P < 0.01.

There were significant improvements in mean BCVA, contrast VA, FVA score, CRT, CCT, MV, and the choroidal area at both 6 and 12 months compared with baseline data (Table 3, Fig. 1a–g). An improvement in the mean BCVA was first observed at 2 months, followed by 4, 6, 8, and 12 months (Fig. 1a). Compared with baseline data, the mean BCVA improvement at 2 months after initial injection that corresponded to the time point after the 3-monthly loading dose was −0.07 ± 0.01 in logMAR (1.9 ± 0.58 letters when each data were adapted to the ETDRS score), 6 months was −0.05 ± 0.02 (2.5 ± 0.79 letters), and 12 months was −0.03 ± 1.97 (1.39 ± 1.67 letters). Improvements in the mean CVA (Fig. 1b) and FVA (Fig. 1c) were first observed at 3 months, followed by 6 and 12 months. A decrease in mean CRT was recorded soon after initial treatment and lasted throughout the treatment course until 12 months (Fig. 1d). A dry macula with no exudative changes confirmed by fundus findings and OCT images was obtained in 80% of the eyes at 2 months; thus, the other 20% of eyes still had exudative changes after 3 loading doses. A dry macula was observed in 66% of the eyes at 6 months and 71% of the eyes at 12 months (Fig. 1h). The mean visual maintenance ratio (VMR) measured by the FVA system and the mean greatest linear dimension (GLD) remained constant throughout the study period. Intraocular pressure (IOP) showed a decrease at 6 months but not at 12 months. The mean NEI-VFQ-25 score improved at both 6 and 12 months compared with the baseline score. The scores for general vision improved at 6 months, while those for general health, ocular pain, distance activity, mental health, role difficulties, and peripheral vision improved at 12 months.

**Table 3:** Mean data at baseline, month 6, and 12.

	Baseline	Month 6	P-value^a^	Month12	P-value^b^
BCVA	0.07 ± 0.09	0.03 ± 0.10	0.020*	0.04 ± 0.12	0.039*
Contrast VA	0.57 ± 0.16	0.47 ± 0.14	0.002**	0.44 ± 0.14	< 0.001**
FVA score	0.37 ± 0.17	0.28 ± 0.18	0.002**	0.29 ± 0.16	0.006**
VMR	0.82 ± 0.09	0.82 ± 0.08	0.670	0.83 ± 0.07	0.650
CRT (μm)	350.7 ± 111.7	252.2 ± 78.3	<0.001**	254.5 ± 87.2	<0.001**
CCT (μm)	248.7 ± 101.8	228.9 ± 79.5	0.002**	226.5 ± 82.3	<0.001**
MV (mm^3^)					
Whole Layer (ILM-BM)	8.90 ± 0.59	8.37 ± 0.56	<0.001**	8.39 ± 0.62	<0.001**
Inner Layer (ILM-INL)	3.92 ± 0.40	3.66 ± 0.42	<0.001**	3.69 ± 0.46	0.002**
Outer Layer (OPL-BM)	5.08 ± 0.37	4.70 ± 0.24	<0.001**	4.70 ± 0.27	<0.001**
Choroidal area (mm^2^)					
Whole area	1.54 ± 0.63	1.39 ± 0.59	<0.001**	1.41 ± 0.61	<0.001**
Luminal area	1.01 ± 0.43	0.91 ± 0.41	<0.001**	0.93 ± 0.41	<0.001**
Stromal area	0.53 ± 0.21	0.48 ± 0.18	<0.001**	0.49 ± 0.21	0.004**
GLD (mm)	3.8 ± 1.5	3.6 ± 1.4	0.210	3.8 ± 1.4	0.730
IOP (mmHg)	14.1 ± 2.8	12.4 ± 2.2	0.003**	13.6 ± 2.4	0.095
VFQ-25 score	75.6 ± 11.9	80.9 ± 9.1	0.014*	84.3 ± 8.1	0.002**

Data are shown in mean ± standard deviation. Wilcoxon signed-rank test was performed. BCVA, best-corrected visual acuity; FVA, functional visual acuity; VMR, visual maintenance ratio; CRT, central retinal thickness; CCT, central choroidal thickness; GLD, greatest linear dimension; MV, macular volume; RPE, retinal pigment epithelium; BM, Bruch’s membrane; IOP, intraocular pressure; VFQ, visual function questionnaire. a, comparison between values at baseline and 6 months; b, comparison between values at baseline and 12 months. *P < 0.05, **p < 0.01.

For each patient, there was no correlation between the improvement in BCVA and decrease in CRT at any time point. However, there was a significant correlation between the improvement in BCVA and decrease in the outer layer MV 6 months after treatment (Table 4). Moreover, the improvements in contrast VA and the FVA score were correlated with the decrease in the inner layer, outer layer, and whole layer MV (Table 4). The improvement in the NEI-VFQ-25 score was correlated with the decrease in CRT at both time points (Table 4).

**Table 4:** Correlation between reduction in retinal values and improvement of visual function

	BCVA		Contrast VA		FVA score		VFQ-25 score	
**Month 6**	r	P-value	r	P-value	r	P-value	r	P-value
CRT (μm)	0.264	0.664	0.497	0.029*	0.242	0.856	−0.577	0.013*
MV (mm^3^)								
Whole Layer (ILM-BM)	0.381	0.164	0.637	0.001**	0.559	0.008**	−0.251	0.944
Inner Layer (ILM-INL)	0.200	0.999	0.545	0.011*	0.524	0.017*	−0.222	0.999
Outer Layer (OPL-BM)	0.479	0.032*	0.611	0.002**	0.558	0.008**	−0.163	0.999
**Month 12**								
CRT (μm)	0.256	0.752	0.265	0.728	0.118	0.999	−0.660	0.004**
MV (mm^3^)								
Whole Layer (ILM-BM)	0.310	0.440	0.562	0.009**	0.544	0.013*	−0.507	0.064
Inner Layer (ILM-INL)	0.163	0.999	0.564	0.009**	0.424	0.110	−0.363	0.388
Outer Layer (OPL-BM)	0.384	0.176	0.499	0.032*	0.474	0.049*	−0.448	0.152

Spearman’s test with Bonferroni correction was performed. Correlation between reduction in retinal layer volumes at month 6 and 12 from baseline values, and improvement of visual function at respective time point was analyzed. BCVA, best-corrected visual acuity; FVA, functional visual acuity; VFQ, visual function questionnaire; CRT, central retinal thickness; MV, macular volume; RPE, retinal pigment epithelium; BM, Bruch’s membrane. *P < 0.05, **p < 0.01.

Subsequently, we analyzed correlations between retinal parameters and choroidal parameters such as central choroidal thickness (CCT), whole choroidal area, choroidal luminal area, and choroidal stromal area, and found that CRT was not correlated with any choroidal parameters (Table 5). However, whole layer MV and outer layer MV were correlated with the choroidal stromal areas at 6 months, and CCT and the whole, luminal, and stromal choroidal areas at 12 months after initial treatment (Table 5). During the study period, the ratio between the luminal and stromal areas remained unchanged (data not shown).

**Table 5:** Correlation between in retinal and choroidal values.

	CCT (μm)		Whole choroidal area (mm^2^)		Choroidal luminal area (mm^2^)		Choroidal stromal area (mm^2^)	
**Baseline**	r	P-value	r	P-value	r	P-value	r	P-value
CRT (μm)	0.069	0.999	0.080	0.999	0.011	0.999	0.253	0.999
MV (mm^3^)								
Whole Layer (ILM-BM)	0.214	0.999	0.238	0.856	0.211	0.999	0.306	0.428
Inner Layer (ILM-INL)	0.078	0.999	0.137	0.999	0.157	0.999	0.157	0.999
Outer Layer (OPL-BM)	0.197	0.999	0.215	0.999	0.144	0.999	0.345	0.268
**Month 6**								
CRT (μm)	0.292	0.496	0.188	0.999	0.180	0.999	0.254	0.512
MV (mm^3^)								
Whole Layer (ILM-BM)	0.443	0.065	0.509	0.019*	0.494	0.024*	0.583	0.004**
Inner Layer (ILM-INL)	0.262	0.680	0.321	0.356	0.331	0.316	0.392	0.142
Outer Layer (OPL-BM)	0.427	0.084	0.448	0.059	0.414	0.102	0.494	0.024*
**Month 12**								
CRT (μm)	0.244	0.848	0.183	0.999	0.161	0.999	0.200	0.999
MV (mm^3^)								
Whole Layer (ILM-BM)	0.511	0.022*	0.527	0.016*	0.516	0.020*	0.510	0.022*
Inner Layer (ILM-INL)	0.398	0.144	0.418	0.108	0.423	0.100	0.383	0.176
Outer Layer (OPL-BM)	0.487	0.032*	0.482	0.036*	0.471	0.044*	0.472	0.044*

Spearman’s test with Bonferroni correction was performed. Correlations between respective retinal and choroidal values at baseline, month 6 and 12 were analyzed. CRT, central retinal thickness; MV, macular volume; RPE, retinal pigment epithelium; BM, Bruch’s membrane; CCT, choroidal thickness. *P < 0.05, **p < 0.01.

No ocular and systemic adverse events were observed in any eye except one (3%) that developed epiphora. This patient dropped out after the 11-month visit due to this symptom (Table 6).

**Table 6:** Adverse events (eyes [%]).

Systemic events	
Any APTC ATEs	0 (0)
Vasucular death	0 (0)
Nonfatal myocardial infarction	0 (0)
Nonfatal stroke	0 (0)
Venous thromboembolic events	0 (0)
Congestive heart failure	0 (0)
**Ocular events**	
Endophthalmitis	0 (0)
Retinal detachment	0 (0)
RPE tears	0 (0)
Submacular hemorrhage	0 (0)
Uveitis	0 (0)
Epiphora	1 (3)

APTC ATE, Antiplatelet Trialists’ Collaboration arterial thromboembolic events; RPE, retinal pigment epithelium.

## Discussion

In the present study, we prospectively evaluated the clinical outcomes of IVA monotherapy administered according to the 2Q8 regimen in 29 eyes of 29 patients with AMD who exhibited a good baseline BCVA (better than 0.6 according to the Landolt C chart and better than 74 letters on the ETDRS VA chart). In addition to an improvement in mean BCVA measured using the conventional Landolt C chart and mean CRT, improvements in mean contrast VA, FVA score, CCT, MV, and choroidal area were observed at 6 months after initial treatment, and were still observed at 12 months. The improvement in the contrast VA and FVA score was correlated with the decrease in MV, which was correlated with CCT and the choroidal area 12 months after initial treatment. An improvement in the NEI-VFQ-25 score was also observed; this was correlated with the decrease in CRT.

BCVA improvement after IVA treatment for AMD patients has been reported in the VIEW1/2 clinical trial^4^, where the baseline BCVA exhibited relatively low values and ranged from 73 to 25 letters on the ETDRS VA chart. In the current prospective study, the benefits of IVA treatment were clarified for patients with a better baseline BCVA; a much better BCVA, compared with baseline, was achieved using IVA treatment, although it remained unclear whether the effect would be evident owing to the possibility of the ceiling effect. A significant improvement in BCVA was first observed at 2 months after initial IVA in the present study, suggesting that three initial monthly injections should be recommended for patients with not only a poor baseline BCVA^4^ but also a good baseline BCVA^22^.

In fact, an improvement at 6 and 12 months after initial treatment was observed in BCVA measured using the conventional Landolt C chart, contrast VA chart, and FVA score. However, if we limited participants to those with a BCVA better than 0.8 (better than 80 letters) at baseline, there was no improvement in BCVA measured using the conventional Landolt C chart (*P* = 0.15); only contrast VA (*P* < 0.001) and FVA score (*P* = 0.04; data not shown) showed an improvement. This finding indicated the possibility of using contrast VA and the FVA system for the evaluation of treatment effects in patients with a very good BCVA at baseline.

A decrease in CRT was first evident after initial treatment and reached a maximum after the third injection, indicating that the impact of the three initial monthly injections was obvious even with regard to CRT.

A dry macula was confirmed just after the IVA injection at 2 and 6 months, whereas that at 12 months was confirmed after a 2-month interval from the last injection. Two previous studies have shown that the ratio after 12 months of treat and extend (TAE) treatment with IVA^23^ and three monthly IVA injections^24^ was 88%. The ratio in the present study was relatively lower than that in the above mentioned retrospective studies, which involved patients with any BCVA value at baseline^23,24^. We believe that the difference in results may have been due to differences in the treatment protocol or follow-up period, not the condition at baseline. Alternatively, the criteria for a dry macula could have played a role; we excluded patients with hyper-reflective foci around the ellipsoid zone and those with no obvious subretinal or intraretinal fluid.

Pro Re Nata^14–16^ or TAE^23^ regimens, rather than continuous monthly or bimonthly injections, tend to be used in daily clinical practice. In such cases, the requirement for another injection is decided by the presence of any exudative fluid. Horizontal and vertical cross-sectional OCT images are routinely obtained in clinical practice; however, we found no correlation between the improvement in BCVA and decrease in CRT observed on cross-sectional OCT images. Interestingly, the improvement in BCVA was correlated with a decrease in MV, particularly the outer layer MV, which involves photoreceptors. Assessment using cross-sectional OCT images alone could result in the overlooking of subtle changes that may affect the prognosis of patients with smaller changes in retinal structures originally.

Moreover, not only the outer layer MV but also the inner layer MV correlated with contrast VA and the FVA score. The inner layer processes the visual input from photoreceptors with or without bidirectional paths in the retinal network^25^, indicating a possibility that contrast VA and the FVA score may involve spatial recognition of visual information, although further research is required to clarify this. It would be suitable to monitor the macular condition with focus on MV in addition to the foveal condition represented by CRT, for optimal outcomes, at least in patients with a relatively good baseline condition.

Of note, MV was not correlated with choroidal area before treatment; however, it gradually correlated after treatment. The outer and whole MV was first correlated with choroidal stromal area, followed by the choroidal luminal and whole choroidal areas. This suggests that an improvement in choroidal stromal condition first affects the retina, in particular, the outer layer, and the relationship between the retinal and choroidal conditions may be normalized by the treatment. In addition, the ratio of the luminal area and the choroidal area remained unchanged after treatment (data not shown), suggesting that the choroidal vessel diameter is correlated with hyperpermeability and the resulting stromal area, regardless of treatment. IVA treatment ameliorated vessel dilatation, which resulted in a decrease in hyperpermeability and exudative changes and a subsequent decrease in the stromal area.

In the present study, all patients exhibited a BCVA that was better than 0.6 in the affected eye, and 90% of patients exhibited a BCVA that was better than 0.8 in the contralateral eye. Accordingly, they did not appear to experience severe difficulties in their activities of daily living (ADL). However, the NEI-VFQ-25 score improved after treatment, suggesting that patients with a good BCVA achieved better practical vision after treatment, and that the treatment had at least satisfied their needs.

Adverse events included epiphora in one patient, although the relationship between IVA treatment and this symptom was not clear. The absence of other ocular and systemic adverse events indicated the safety of this treatment in the present study.

The limitations of this study include the relatively small number of participants with various subtypes including typical AMD, PCV, and RAP, the inclusion of a single treatment arm, the lack of comparison with other treatment regimens, and the relatively short follow-up period. However, as demonstrated in a control group included in clinical trials of photodynamic therapy^26^ and pegaptanib^27^, AMD patients cannot maintain visual function compared with baseline data without any treatments, and the impact of the results in the current study was obvious. Although the NEI-VFQ-25 is an established method, it is subjective, and the scores may also include the satisfaction of completing the treatment regimen.

In conclusion, IVA treatment according to the 2Q8 regimen for patients with wet AMD exhibiting a good BCVA at baseline improved BCVA, retinal condition, and QOL evaluated by NEI-VFQ-25 at 6 months; these effects lasted until 12 months after the initial treatment. The information obtained by the prospective study was firstly provided in the current study, and will be valuable for daily practice; it will support the clinicians’ confidence in deciding and recommending the application of this treatment for patients with good BCVA. Currently, more patients are visiting the clinic before their BCVA becomes substantially decreased. Compared to the BCVA measured using the conventional Landolt C chart, the contrast VA and FVA scores were useful for assessing subtle changes in visual function, whereas the MV and choroidal area on OCT images, rather than CRT, were useful for assessing minimal changes of retinal condition related to visual function in the patients with good BCVA before the initial treatment. Although further research is required, this study demonstrates the efficacy and benefits of IVA treatment for patients with AMD who exhibit a good BCVA at baseline.

## Methods

This prospective, single-arm intervention study was conducted in accordance with the tenets of the Declaration of Helsinki and was approved by the Ethics Committee of the Keio University School of Medicine (20130164). It is registered under the number UMIN000012221 (November 6 2013). Written informed consent was obtained from all subjects before study initiation. The datasets generated during and/or analyzed during the current study are not publicly available but are available from the corresponding author on reasonable request.

### Study Participants

In total, 29 eyes of 29 patients with a BCVA better than 0.6 when measured using the conventional Landolt C chart (<0.22 logMAR; better than 74 letters on the ETDRS chart) who were diagnosed with AMD at the Medical Retina Division Clinic (AMD Clinic) of the Department of Ophthalmology, Keio University Hospital (Tokyo, Japan) between November 2013 and June 2015 were included. All included patients had exudative changes, were naïve to the treatment, and attended the clinic for at least 12 months, during which IVA was the only treatment they received. No patients with early AMD without any exudative changes at baseline were included.

### Ophthalmological examinations

We performed comprehensive ophthalmological examinations, including BCVA measurements using the conventional Landolt C chart, contrast VA chart (CSV-1000 LanC charts; VectorVision, Inc., Greenville, OH, USA)^9^, and FVA system (FVA system, AS-28 FVA measurement system; Kowa, Tokyo, Japan);^9,28^ slit lamp biomicroscopy; IOP measurements; and binocular indirect ophthalmoscopy after pupil dilation with 0.5% tropicamide at particular time points (Table 1) for all subjects. The outcome values for the FVA measurement system were the FVA score, average VA measured during the 60-s period, and VMR calculated as follows: VMR = (lowest logMAR VA score − FVA at 60 s)/(lowest logMAR VA score − baseline VA).

### Angiographies

We performed fluorescein angiography and indocyanine green angiography and obtained fundus photographs using the Topcon TRC 50DX retinal camera (Topcon Corporation, Tokyo, Japan). For all eyes, experienced AMD specialists (SM, NN, and YO) performed image reading, and measured the GLD.

### OCT

We obtained OCT images using the Heidelberg Spectralis OCT system (Heidelberg Engineering GmbH, Dossenheim, Germany), and evaluated CRT, CCT, MV, and choroidal area. MV in each area with a 6-mm diameter, as determined in ETDRS^29^, was measured using the version 6 OCT software (Heidelberg Engineering GmbH, Dossenheim, Germany). The inner layer MV was defined from the inner limiting membrane (ILM) to inner nuclear layer (INL), the outer layer MV was from the outer plexiform layer (OPL) to Bruch’s membrane (BM), and the whole layer MV was from ILM to BM. For lesion areas where autosegmentation was not precise, we made manual adjustments as previously described^30^. The choroidal area was measured using a modification of the binarization method for OCT images reported by Sonoda *et al*.^31,32^. Briefly, subfoveal choroidal images recorded by enhanced-depth imaging and 6-mm distance areas determined by ETDRS (3-mm distance from the fovea to each side) were analyzed and averaged. The luminal and stromal areas were converted to binary images using the Niblack method and measured using ImageJ software (developed by Wayne Rasband, National Institutes of Health, Bethesda, MD, USA; available at http://rsb.info.nih.gov/ij/index.html).

### NEI-VFQ-25

For the evaluation of QOL, all patients were asked to complete the Japanese version of the NEI-VFQ-25 (Ver 1.4; iHOPE International Inc., Kyoto, Japan).

### IVA monotherapy and follow-up

IVA (2 mg, 0.05 mL) injections were administered under sterile conditions once a month for 3 months and every 2 months thereafter (2Q8 regimen). Patients were followed up every month, within 7 days after injection.

### Statistical analyses

The primary endpoint was a decrease in exudative fluid at 6 months, while the secondary endpoint was a change in fundus findings, including exudative fluid, and visual outcomes at 12 months after initial treatment. One patient dropped out after the visit at 11 months after initial treatment; therefore, his data for month 12 was not included in our analyses. All statistical analyses were performed using commercially available software (SPSS, v.23.0; IBM Japan, Tokyo, Japan). Baseline data were compared with those obtained at 6 and 12 months using Wilcoxon signed-rank tests or Spearman’s tests with Bonferroni correction. A *P*-value of <0.05 was considered statistically significant.
